# Hepatic carcinosarcoma: a rare and aggressive case with unusual molecular signature!

**DOI:** 10.3389/fonc.2026.1759219

**Published:** 2026-01-30

**Authors:** Sayed Ali Almahari, Maryam Al-Ani, Abed M Zaitoun, Gregory Gordon, Samiya Ibrahim, Arvind Arora, Bahaaeldin Baraka

**Affiliations:** 1 Department of Cellular Pathology, Nottingham University Hospitals NHS Trust, Queen’s Medical Center, Nottingham, United Kingdom; 2 Department of Oncology, Nottingham University Hospitals NHS Trust, City Hospital, Nottingham, United Kingdom; 3 Nottingham Digestive Diseases Centre, Division of Translational Medical Sciences, School of Medicine, University of Nottingham, Queen’s Medical Centre, Nottingham, United Kingdom; 4 National Institute for Health Research Nottingham Biomedical Research Centre, Nottingham University Hospitals NHS Trust and University of Nottingham, Queen’s Medical Centre, Nottingham, United Kingdom; 5 Department of Hepatobiliary Surgery, Nottingham University Hospitals NHS Trust, Queen’s Medical Center, Nottingham, United Kingdom

**Keywords:** cholangiocarcinoma, hepatic carcinosarcoma, KIAA1549::BRAF fusion, TERT promoter mutation, TP53 mutation

## Abstract

**Background:**

Primary hepatic carcinosarcoma is a rare, aggressive tumour with both carcinomatous and sarcomatous components. Limited documented cases hinder comprehensive understanding, making diagnosis, treatment, and management particularly challenging for clinicians.

**Case:**

A 62-year-old female, with prior cervical squamous cell carcinoma 7 years ago, underwent right hemihepatectomy, cholecystectomy, and diaphragmatic resection in 2025. Grossly, a 180 mm white hepatic tumour with a large cystic cavity was seen adherent to the diaphragm, extending to the resection margin (R2), while the hepatic resection margin was clear (R0). Histology confirmed hepatic carcinosarcoma (pT4), comprising cholangiocarcinoma (CK7, BerEP4+), hepatocellular carcinoma (Glypican-3+), and squamous carcinoma (p63, p40+). Sarcomatous areas included rhabdomyosarcomatous (Desmin, Myogenin, MyoD1+), leiomyosarcomatous (SMA+), and chondrosarcomatous (S100+) differentiation. PLAP and CD117 positivity suggested germ cell-like features. There is no distinct separation between the carcinomatous and sarcomatous components. Molecular profiling revealed a KIAA1549::BRAF fusion alongside oncogenic variants: TERT c.-124C>T (VAF 0.60) and TP53 c.811G>A p. (Glu271Lys) (VAF 0.90). Targeted panel sequencing showed no other actionable mutations. MSI readout was 2.5%, confirming microsatellite stability (MSS).

**Outcome:**

Multidisciplinary review at tertiary centres confirmed the rarity and grave outlook. The patient developed early recurrence with thoraco-abdominal deposits, venous thromboembolism, and pleural effusion. Paclitaxel–carboplatin chemotherapy was commenced with dose modifications for hepatotoxicity, complicated by infusion reactions, mild neuropathy, and mucositis.

**Conclusion:**

This case underlines the extreme morphological and molecular heterogeneity of hepatic carcinosarcomas, the rapid progression despite surgery, and the limited systemic treatment options available for such rare tumours.

## Introduction

Hepatic carcinosarcomas are exceptionally rare and highly aggressive primary liver tumors, representing approximately 0.3–1% of all hepatic malignancies (1, 2). They are characterized by a distinctive biphasic morphology composed of both epithelial (carcinomatous) and mesenchymal (sarcomatous) components (3). The epithelial component is most often a hepatocellular carcinoma or cholangiocarcinoma, whereas the mesenchymal counterpart may exhibit spindle cell, chondroid, osteoid, rhabdoid, or other heterologous differentiation (4).

This biphasic histology is not merely a diagnostic hallmark but also underlies the tumor’s aggressive biological behavior and the considerable therapeutic challenges it poses. Patients typically present with large, rapidly growing hepatic masses and nonspecific systemic symptoms, often leading to diagnosis at an advanced stage (5). Due to its rarity, current understanding of hepatic carcinosarcoma relies largely on isolated case reports and small retrospective series, with no established diagnostic or therapeutic consensus (2, 6).

Here, we describe a case of hepatic carcinosarcoma with complex epithelial, squamous, and sarcomatous differentiation, harboring a novel KIAA1549::BRAF fusion alongside TERT and TP53 mutations. This case highlights the diagnostic intricacies, distinctive molecular alterations, and formidable management challenges associated with this rare malignancy.

## Case presentation

**Figure 1:**
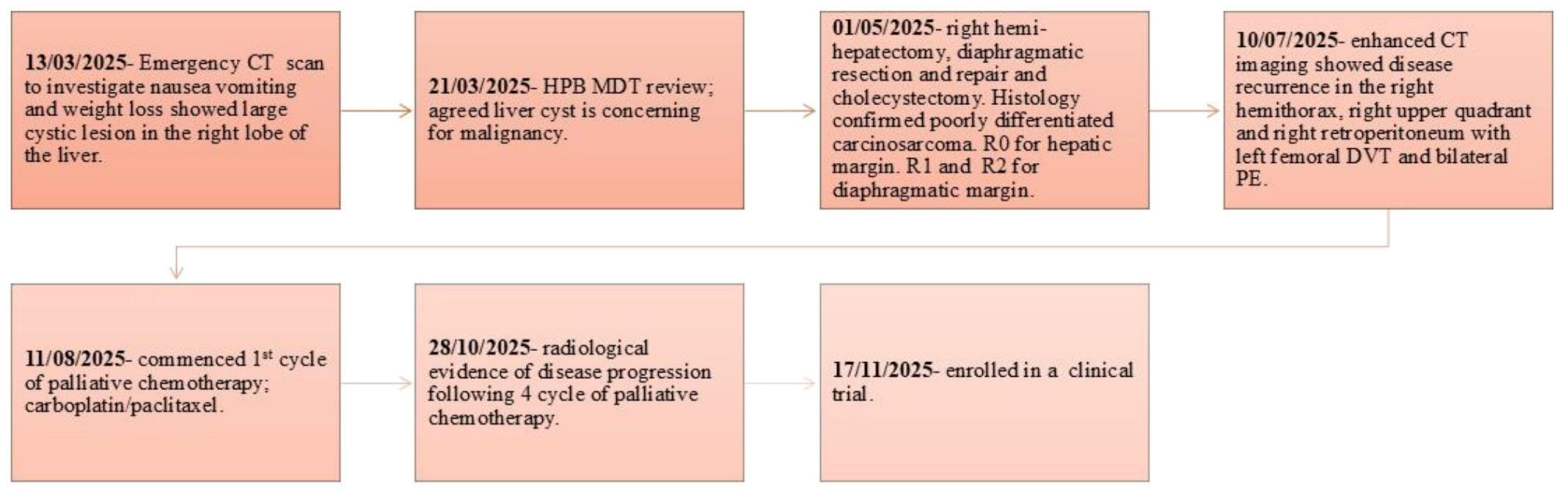
Summary of clinical events from diagnosis until disease progression following palliative chemotherapy. CT, computerised tomography; HPB, Hepato-pancreato-biliary; MDT, multidisciplinary team; R0, clear resection margin; R1 and R2, resection margin positive microscopically (R1) and macroscopically (R2). DVT, deep vein thrombosis; PE, pulmonary embolism.

**Figure 2:**
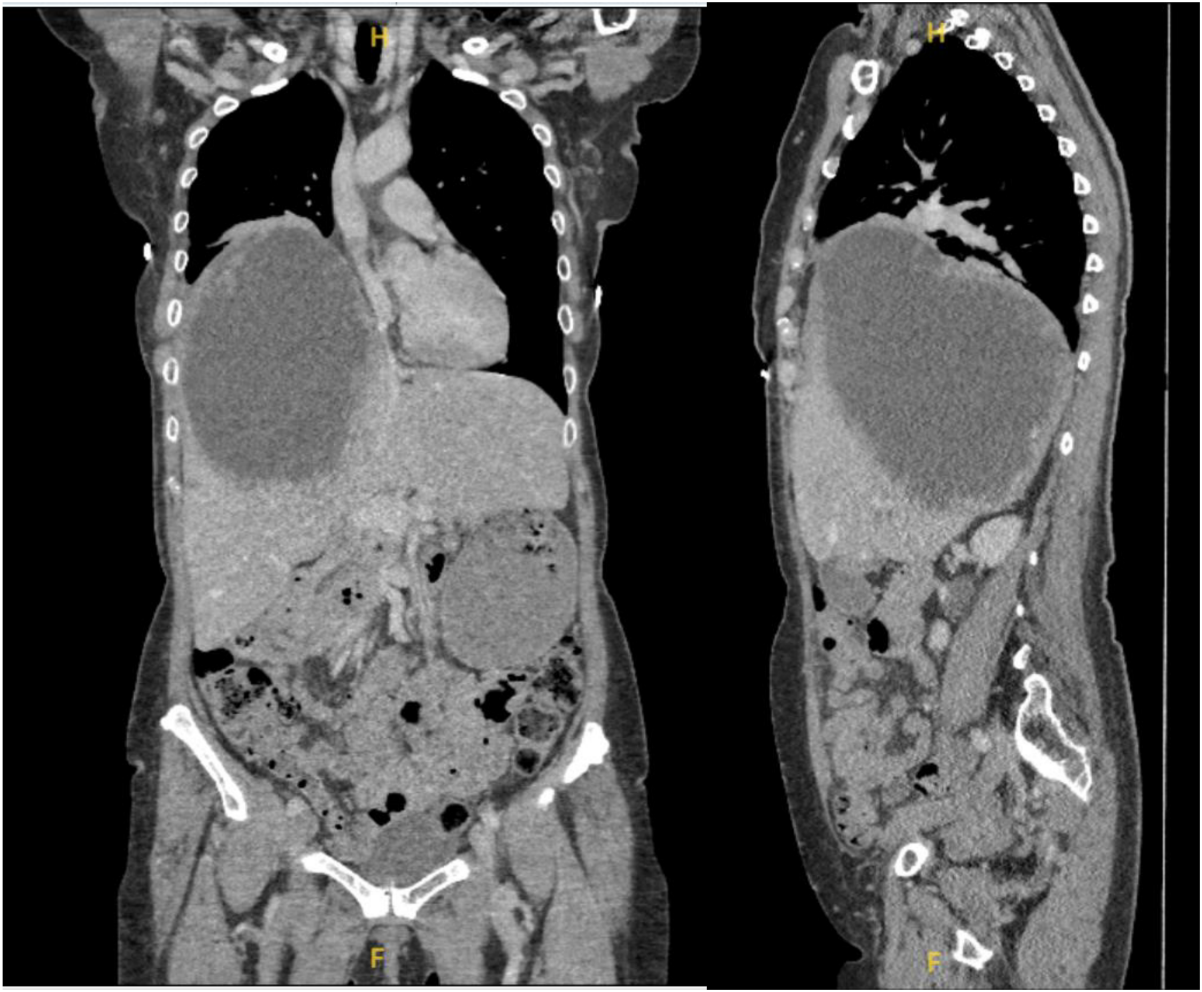
Preoperative imaging of the tumor. Sagittal and coronal CT scans demonstrating the extent of the hepatic tumor.

A 62-year-old female, a known case of stage IA1 squamous cell carcinoma (SCC) of the cervix diagnosed seven years earlier, for which she underwent total abdominal hysterectomy and bilateral salpingo-oophorectomy, presented with a two-month history of nausea and unintentional weight loss of 20 kg (**Figure 1**). On physical examination, a large right upper quadrant abdominal mass was palpable. Computed tomography (CT) of the abdomen revealed a 19 cm cystic lesion occupying the right hepatic lobe, displacing the right hemidiaphragm superiorly and extending above the level of the aortic arch (**Figure 2**).

The patient was discussed at HPB MDT, which assessed that, as the cystic lesion had solid components and wall enhancement on imaging, it was concerning for a cystadenocarcinoma. Surgery was recommended by MDT and to the patient subsequently in the clinic.

The surgical resection was performed through a reverse-L incision in the right upper quadrant. Owing to the tumor’s size, extent, and associated adhesions, the liver could not be mobilized. Consequently, the surgeon employed a liver-hanging maneuver to separate the right liver from the inferior vena cava and the left liver, followed by a medial-to-lateral resection. A portion of the diaphragm was resected en bloc with the specimen; however, in view of the patient’s borderline fitness, an extensive diaphragmatic resection was not undertaken.

Postoperatively, the patient recovered well. Apart from transient derangement of liver function tests and a small pleural effusion, the postoperative course was uneventful, and the patient was discharged on day 12.

Initially, fluid aspirated from the cyst was submitted for cytological examination. The smears showed a necrotic background containing rare malignant-appearing epithelial cells. Then two days later, we received the right hemihepatectomy specimen.

**Figure 3:**
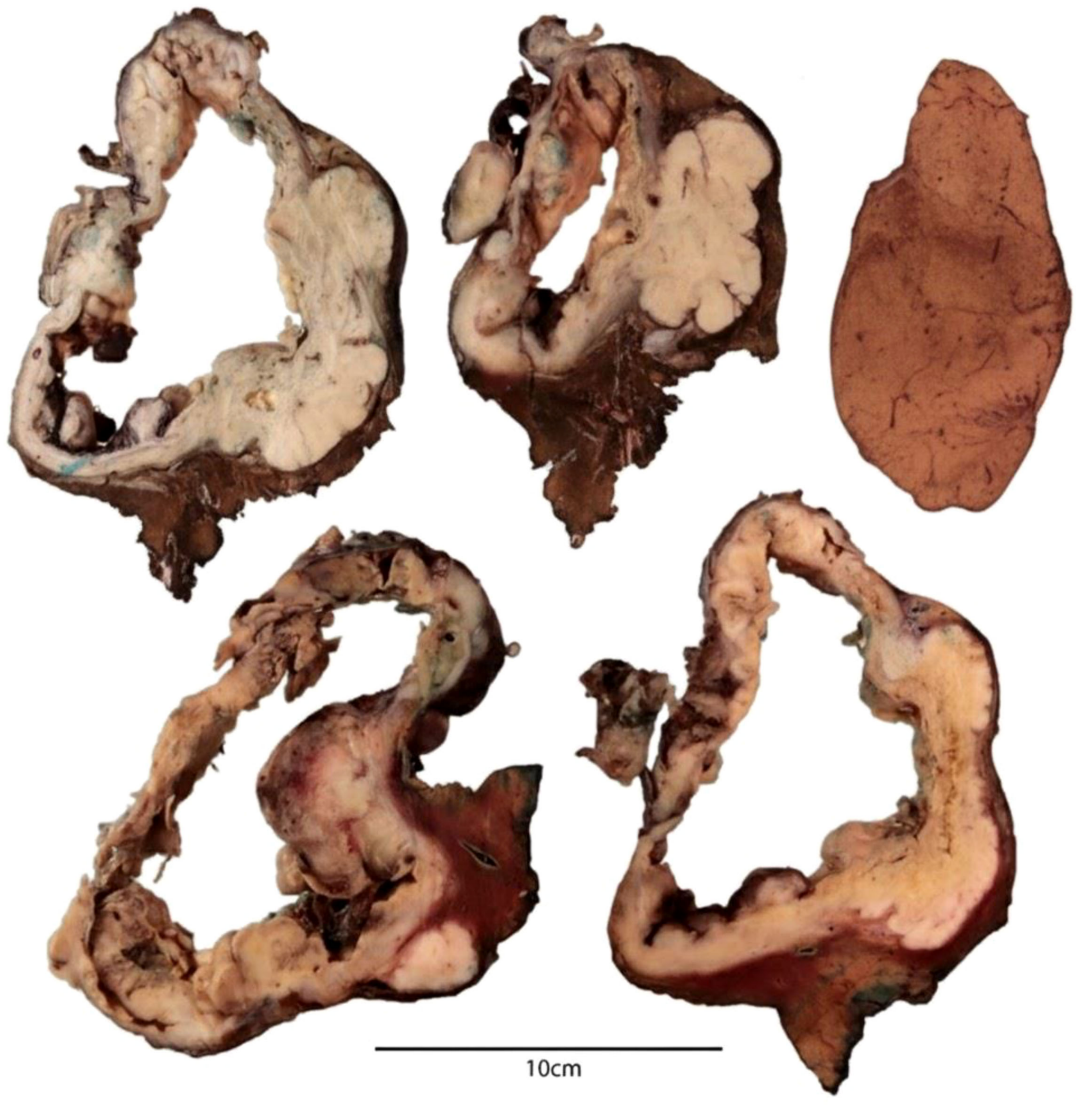
Gross features of the hepatic cyst. The cut surface shows a unilocular cyst with a thickened wall, displaying areas of necrosis and myxoid change.

Gross examination of the resected specimen revealed a large cystic lesion with a thickened, heterogeneous wall. The cut surface showed myxoid-like areas and foci of necrosis (**Figure 3**). The tumor infiltrated the overlying diaphragm and reached the diaphragmatic resection margin, while the hepatic parenchymal margin was free of tumor.

**Figure 4:**
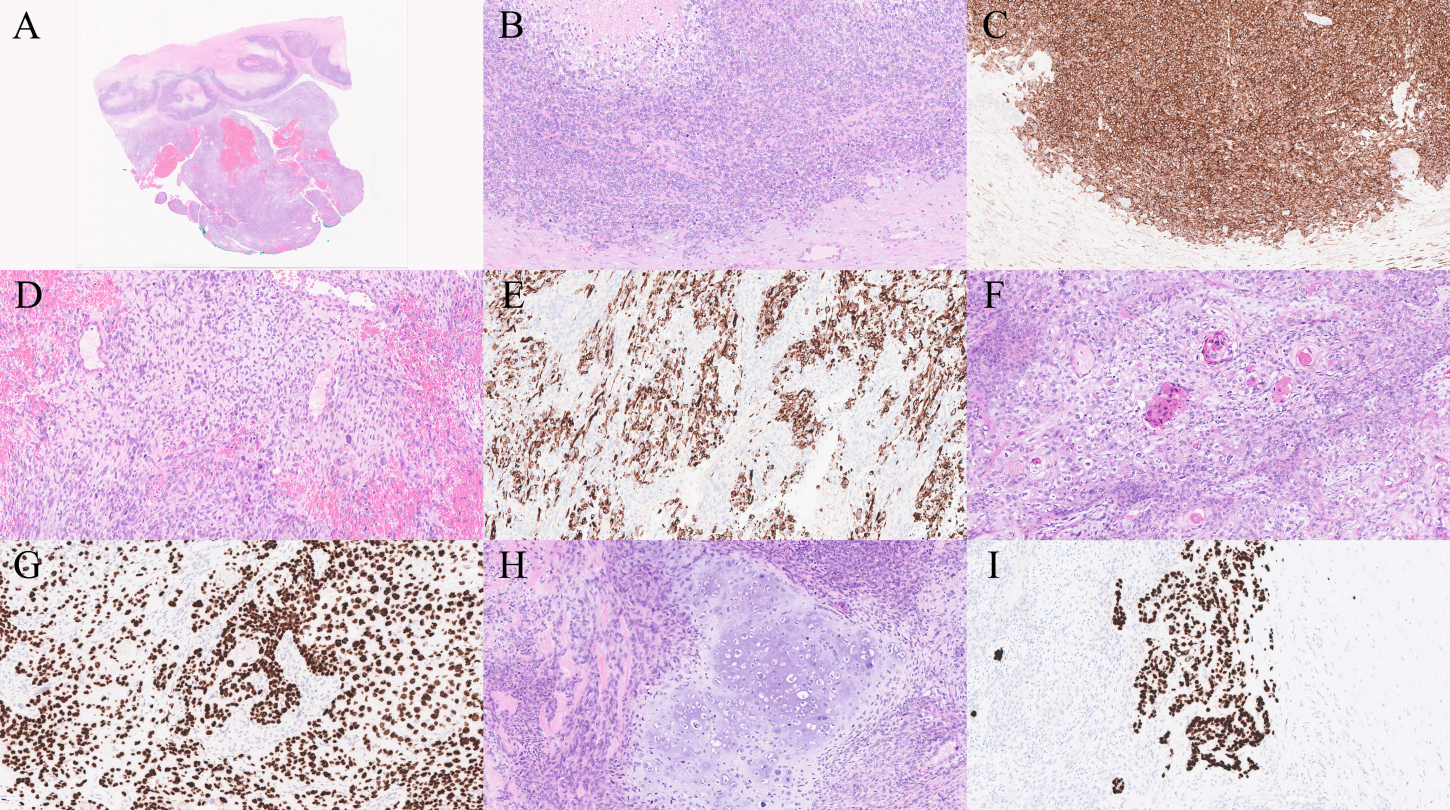
**(A)** Low-power view showing two distinct components: sheets of blue cells with necrosis (upper area) and a spindle cell component (lower area) (H&E ×1). **(B, C)** Intermediate-power view showing a poorly differentiated cholangiocarcinoma component composed of round epithelioid cells, demonstrating CK7 positivity (H&E ×20, CK7 ×20). **(D, E)** Intermediate-power view showing a leiomyosarcomatous component showing spindle cells with scattered highly atypical forms and SMA positivity (H&E ×20, SMA ×20). **(F, G)** Intermediate-power view showing a moderately differentiated squamous cell carcinoma component with keratinization and P40/P63 positivity (H&E ×20, P40/P63 ×20). **(H, I)** Intermediate-power view showing a chondrosarcomatous component exhibiting atypical cartilaginous differentiation and S100 positivity (H&E ×20, S100 ×20).

Microscopically, the tumour exhibited a biphasic pattern composed of high-grade cholangiocarcinoma and moderately differentiated squamous cell carcinoma. In addition, a prominent sarcomatous component was present, showing heterologous differentiation with leiomyosarcomatous, chondrosarcomatous, and rare rhabdomyosarcomatous areas. Foci resembling yolk sac tumour were identified in one of the sections, suggesting the possibility of divergent differentiation within the tumor (**Figure 4**).

Immunohistochemical studies demonstrated that the cholangiocarcinoma component was positive for CK7 and BerEP4, while β-catenin showed membranous staining without nuclear translocation, indicating the absence of an activating mutation (**Figure 4**). The squamous cell carcinoma component was positive for p63 and p40, confirming squamous differentiation. Immunostains for neuroendocrine markers, including Synaptophysin and Chromogranin A, were negative, excluding neuroendocrine differentiation. AFP staining was negative, ruling out a hepatocellular carcinoma component.

The sarcomatous areas displayed variable immunoprofiles depending on their degree of differentiation. The rhabdomyosarcomatous regions were positive for Desmin, Myogenin, and MyoD1, confirming skeletal muscle differentiation. The leiomyosarcomatous areas were diffusely and faintly positive for smooth muscle actin (SMA), while the chondrosarcomatous areas showed diffuse and faint S100 positivity. In addition, PLAP and CD117 were focally positive in lace-like areas, findings that may suggest a germ cell component. β-hCG staining was negative, thereby excluding choriocarcinomatous differentiation.

**Figure 5:**
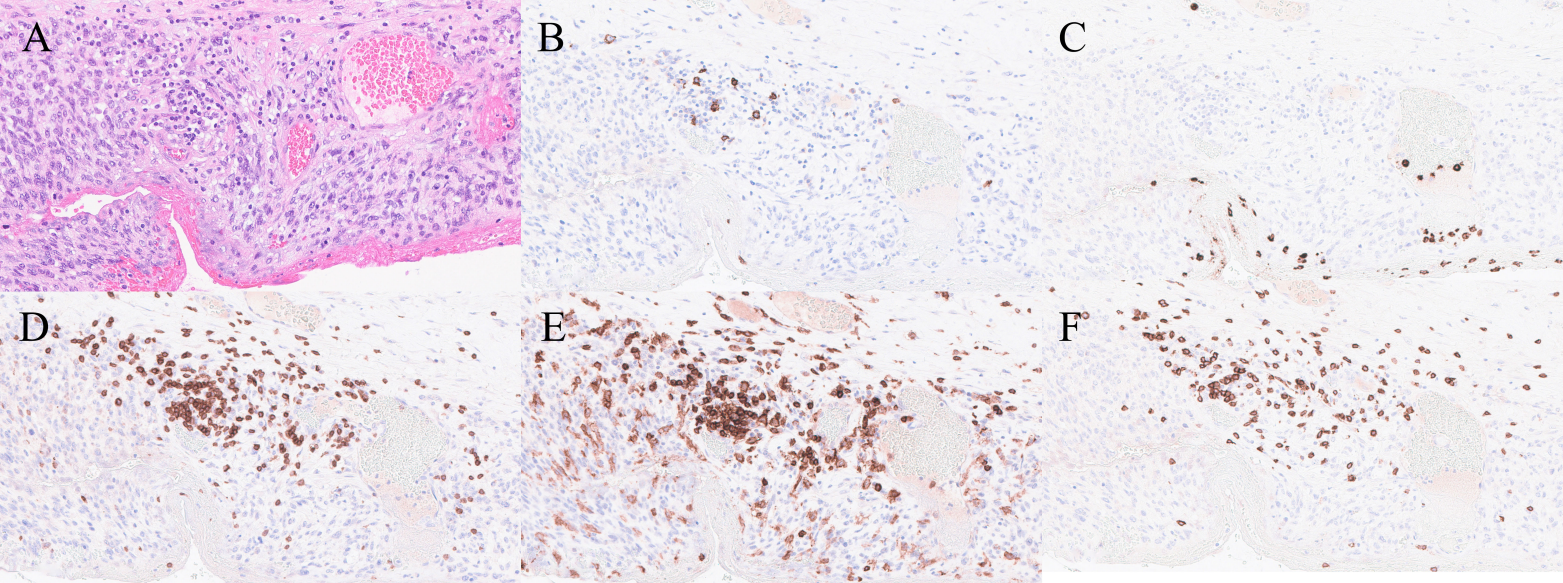
**(A)** High-power view showing the tumor cells with associated inflammatory cells (H&E x40). **(B)** CD20 showing a focal positivity in a few B-cells (CD20 x40). **(C)** CD15 shows scattered polymorphs (CD15 x20). **(D–F)** CD3 shows a predominance of T-cells with a mix up of CD4 cells and CD8 cells (CD3, CD4 and CD8 x40).

Moreover, the tumor-associated inflammatory infiltrate demonstrated a predominant T-lymphocytic population (**Figure 5**). Both intratumoral and peritumoral lymphocytes were largely CD3-positive, with CD8-positive cytotoxic T cells representing most of the T-cell subset, while CD4-positive helper T cells were present in lower proportions. Scattered CD15-positive neutrophils were identified within the tumor stroma. In addition, numerous CD68-positive macrophages were observed (not represented in the figure), particularly concentrated around areas of tumor necrosis.

Molecular profiling was performed to further characterize the tumor. Targeted next-generation sequencing identified a KIAA1549::BRAF fusion, along with oncogenic variants TERT c.-124C>T (VAF 0.60) and TP53 c.811G>A p.(Glu271Lys) (VAF 0.90). No other actionable mutations were detected on the targeted panel. The microsatellite instability (MSI) readout was 2.5%, confirming microsatellite stability (MSS). These molecular findings, in conjunction with the histopathologic and immunophenotypic features, supported a diagnosis of a complex hepatic carcinosarcoma exhibiting cholangiocellular, squamous, sarcomatous, and germ cell–like differentiation.

## Discussion

The overall morphology and immunophenotype support a hepatic carcinosarcoma with high-grade cholangiocarcinoma and squamous cell carcinoma components, accompanied by heterologous mesenchymal differentiation. Such biphasic primary liver tumors are rare, and reported series highlight their diagnostic complexity and aggressive clinical behavior. The prevailing theory suggests divergent differentiation from a common epithelial progenitor rather than a true collision of distinct neoplasms, which is supported by the intimate admixture of epithelial and mesenchymal elements in our case (2).

In establishing this diagnosis, several key differential diagnoses were systematically excluded. Metastatic germ cell tumor was considered because of focal PLAP and CD117 expression and yolk sac–like areas but was ruled out by the absence of a ovarian or mediastinal primary, no clinical history of germ cell neoplasia, and the presence of well-defined malignant epithelial components. Primary hepatic teratoma was excluded based on the patient’s age and the lack of organized derivatives of all three germ layers or mature tissue elements, with the tumor instead showing overt malignant epithelial and mesenchymal differentiation. Metastatic sarcoma with epithelial differentiation was unlikely given the coexistence of immunophenotypically distinct cholangiocarcinoma and squamous cell carcinoma components and the absence of a primary sarcoma elsewhere. Sarcomatoid hepatocellular carcinoma was excluded due to the lack of hepatocellular morphology, negativity for hepatocellular markers, and clear biliary and squamous differentiation. Collectively, these findings support a diagnosis of primary hepatic carcinosarcoma (7).

There are no reported cases of hepatic carcinosarcomas with germ line differentiation. However, there are some reported cases of primary mixed germ cell tumors with sarcomatous components (8), along with cases of cholangiocarcinomas with yolk sac differentiation (9). In both of those scenarios, tumors showing yolk sac differentiation, clinical implications appear to be context dependent: in cholangiocarcinoma with somatic yolk sac differentiation, management and outcome remain driven by the underlying carcinoma and treated with standard biliary chemotherapy, whereas in primary hepatic mixed germ cell tumors the presence of a yolk sac component defines prognosis and mandates germ cell–directed systemic therapy (8, 9).

The tumor microenvironment showed a prominent immune infiltrate dominated by CD3-positive T lymphocytes, with a predominance of CD8-positive cytotoxic T cells. Similar immune profiles have been described in cholangiocarcinoma, where dense CD8-positive infiltrates may reflect an ongoing but functionally impaired anti-tumor immune response (10). The relative paucity of CD4-positive T cells may further contribute to ineffective immune surveillance (11). In addition, the accumulation of CD68-positive macrophages around necrotic tumor areas is consistent with the presence of tumor-associated macrophages, which are known to play a key role in tumor progression, immune modulation, and metastatic potential in liver malignancies (12). Although limited by the descriptive nature of a case report, these findings highlight the complex immune landscape of hepatic carcinosarcoma and support the concept that inflammatory and immune components may contribute to its aggressive behavior.

Molecular profiling revealed a KIAA1549::BRAF fusion together with TERT promoter c.-124C>T and TP53 c.811G>A p.(Glu271Lys) variants in a microsatellite-stable background. The KIAA1549::BRAF fusion results from a 7q34 tandem duplication that removes the N-terminal autoinhibitory domain of BRAF while retaining its kinase domain, producing constitutive MAPK pathway activation. It is most characteristic of pediatric low-grade gliomas but has occasionally been detected in extracranial tumors, including sarcomas and carcinomas of the thyroid and pancreas (13–15). To date, no previous reports describe this fusion in cholangiocarcinoma, hepatic carcinosarcoma, or other primary hepatic epithelial malignancies. BRAF alterations in intrahepatic cholangiocarcinoma are usually point mutations such as V600E; fusions are exceptional and involve other partners such as YWHAZ–BRAF (10). Our finding therefore, appears to represent the first documented instance of BRAF alterations, including both translocation and point mutation in the English literature.

The co-occurring TERT promoter c.-124C>T mutation activates telomerase transcription and is common across cancers but infrequent in intrahepatic cholangiocarcinoma, where it occurs in less than 10% of cases (16). In contrast, TP53 mutations are prevalent in this tumor type and portend an unfavorable prognosis (17). Together, the molecular alterations in our case suggest activation of the MAPK and telomerase pathways, coupled with a defective tumor-suppressor network, driving an aggressive phenotype.

Clinically, our patient underwent surgery, and a postoperative CT scan showed very early disease recurrence, with appearances suggesting metastatic deposits in the right hemithorax, right upper quadrant, and right retroperitoneum, deep vein thrombosis involving the left femoral and iliac veins, together with bilateral pulmonary emboli. She subsequently received paclitaxel–carboplatin chemotherapy with palliative intent but unfortunately demonstrated a poor clinical response. This rapid progression is consistent with the poor overall outcomes typically seen in such aggressive disease, which aligns with the poor outcomes previously reported for hepatic carcinosarcoma and sarcomatous cholangiocarcinoma, which generally recur within months despite adjuvant therapy (2, 6).

From a therapeutic standpoint, tumors harboring BRAF fusions signal through RAS-dependent RAF dimers and often exhibit paradoxical activation when exposed to V600E-specific inhibitors. They may, however, respond to MEK inhibitors or type-II RAF inhibitors such as tovorafenib, recently approved for pediatric low-grade glioma harboring BRAF fusions (18–20). Although data on biliary tract tumors are lacking, the presence of a canonical fusion in this case highlights potential avenues for pathway-targeted therapy if clinically applicable.

## Conclusion

In summary, this case expands the molecular spectrum of hepatic carcinosarcoma by documenting a novel KIAA1549::BRAF fusion in conjunction with TERT and TP53 mutations. The combination of complex histology and aggressive clinical behavior underscores the need for comprehensive molecular testing in rare hepatic malignancies to uncover actionable alterations and improve therapeutic stratification.

## Data Availability

The raw data supporting the conclusions of this article will be made available by the authors, without undue reservation.
